# Interventions to prevent disability in frail community-dwelling elderly: a systematic review

**DOI:** 10.1186/1472-6963-8-278

**Published:** 2008-12-30

**Authors:** Ramon Daniels, Erik van Rossum, Luc de Witte, Gertrudis IJM Kempen, Wim van den Heuvel

**Affiliations:** 1 Faculty of Health and Care, Zuyd University of Applied Sciences, Heerlen, the Netherlands; 2 Centre of Research on Autonomy and Participation, Zuyd University of Applied Sciences, Heerlen, the Netherlands; 3 Centre of Research on Technology in Health Care, Zuyd University of Applied Sciences, Heerlen, the Netherlands; 4 School for Public Health and Primary Care (Caphri), Maastricht University, Maastricht, the Netherlands

## Abstract

**Background:**

There is an interest for intervention studies aiming at the prevention of disability in community-dwelling physically frail older persons, though an overview on their content, methodological quality and effectiveness is lacking.

**Methods:**

A search for clinical trials involved databases PubMed, CINAHL and Cochrane Central Register of Controlled Trials and manually hand searching. Trials that included community-dwelling frail older persons based on physical frailty indicators and used disability measures for outcome evaluation were included. The selection of papers and data-extraction was performed by two independent reviewers. Out of 4602 titles, 10 papers remained that met the inclusion criteria. Of these, 9 were of sufficient methodological quality and concerned 2 nutritional interventions and 8 physical exercise interventions.

**Results:**

No evidence was found for the effect of nutritional interventions on disability measures. The physical exercise interventions involved 2 single-component programs focusing on lower extremity strength and 6 multi-component programs addressing a variety of physical parameters. Out of 8 physical exercise interventions, three reported positive outcomes for disability. There was no evidence for the effect of single lower extremity strength training on disability. Differences between the multi-component interventions in e.g. individualization, duration, intensity and setting hamper the interpretation of the elements that consistently produced successful outcomes.

**Conclusion:**

There is an indication that relatively long-lasting and high-intensive multicomponent exercise programs have a positive effect on ADL and IADL disability for community-living moderate physically frail older persons. Future research into disability prevention in physical frail older persons could be directed to more individualized and comprehensive programs.

## Background

Frail elderly people are at much higher risk for falls, infections, disabilities, hospitalization, institutionalization, and death, compared with their age-matched non-frail counterparts [1-3]. In scenarios that predict future health service delivery in the Western world, the rapid increase in frail elderly is seen as one of the major challenges of health care [4-6]. There is an increasing interest for frail elderly being particularly vulnerable for developing disabilities [7-9]. As disability is closely related to medical spending, it is believed that prevention of disability can lead to reduced health care costs [10]. Current literature supports the notion of frailty as a pathway to disability that is not a direct result of chronic disease, but instead is associated with age-related loss of physical condition and reserve [11,12]. This viewpoint on frailty points to opportunities for interventions aimed at either delaying the onset of frailty or reducing its adverse outcomes [1,4].

A widely accepted definition and clear criteria for frailty are lacking [1,5,13]. Markle-Reid and Brown [5] reported substantial disagreement in the literature how frailty is defined and measured. Based on recent studies into risk-factors for adverse outcomes in frail elderly there appears to be a growing consensus for acknowledging physical frailty as a construct that can be identified by frailty components [14]. Interventions for physical frailty stem from the idea that the causal pathway towards frailty is a negative spiral in which inflammation, neuroendocrine deregulation and sarcopenia play a role implying that interventions can be targeted at physical frailty independent of specific diseases [15]. The Interventions on Frailty Working Group [14] recommended the development and testing of preventive interventions for physically frail elderly based on any of the following screening criteria: mobility, strength, balance, motor processing, nutrition, endurance or physical activity. Over the last decade, several intervention studies aiming at the prevention of disability in the elderly using physical frailty indicators as inclusion criteria have been reported. An overview of the effectiveness and content of these interventions is, however, not available. This systematic review was conducted to assess the content, the methodological quality and the effectiveness of intervention studies for the prevention of disability in community-dwelling physically frail elderly.

## Methods

### Search strategy

On May 16 2007 databases PubMed, the Cochrane Central Register of Controlled Trials (CENTRAL) and CINAHL were searched for randomized- and controlled clinical trials by using "frail*", "vulnerable", "at risk", "high risk", "low functioning", and the MESH terms "chronic disease" and "disabled persons" in combination with the MESH term "aged". Search terms for outcomes focused on disability measures and included terms like "disabil*", "functional decline", "functional capabilit*", "functional performance", "independen*" and MESH terms "activities of daily living", "quality of life" and "well being". To restrict the search to interventions that targeted community-dwelling elderly terms like "home*", "in-home*", "communit*", "independent living" and MESH term "primary care" were added. Additionally studies were searched by hand-searching reference lists from relevant papers. The search was restricted to articles in English, Dutch and German. There was no restriction for type of intervention or year of publication.

### Selection criteria

Clinical trials where community-dwelling frail elderly were the target group were included. Studies had to include frail elderly based on at least one of the physical frailty indicators as described by Ferrucci et al. [14]. Table 1 gives an overview of these indicators. Studies that, for instance, equalized frailty solely on the basis of presence of disabilities, chronic illness, the eligibility of care or discharge from hospital were excluded. As interventions in frail elderly focusing on disability prevention was the aim of this study, the presence of outcome measurement 'disability' was another criteria for inclusion. Disability was defined as experienced difficulty in performing activities in any domain of life [16]. Avlund [17] found that most current studies of disability among older persons focus on the ability to carry out the activities of daily living. The need to focus on activities of daily living as a fundamental outcome is well justified as persons who are disabled in activities of daily living function can not successfully live alone [18]. In this review only studies reporting about measurements on Activities of Daily Living (ADL) or Instrumental Activities of Daily Living (IADL) were included.

**Table 1:** Physical frailty indicators

Indicators*	**Possible measures**^**†**^
Mobility	Gait speed

Strength	Grip strength
	Chair rise
	Knee extensor strength

Endurance	Lack of energy
	Tiredness
	Oxygen-uptake

Nutrition	Under-nutrition (decreased food intake)
	Weight loss
	Body Mass Index
	Obesity

Physical inactivity	Frequency and duration of walking and bicycling in the previous week and the average amount of time spent monthly on hobbies, gardening, odd jobs, and sports

Balance	Items from Berg Balance Scale like
	Sitting to standing
	Standing to sitting
	Standing unsupported

Motor processing	Coordination
	Movement planning
	Movement speed

* As mentioned in Ferrucci et al. [14]

^† ^As reported in frailty literature

### Data extraction and analysis

A first selection of relevant studies was made on title-level using a conservative approach, meaning that in case of doubt an article would always be screened on abstract-level. The second (abstract-level) and third selection phase (full-text level) were independently undertaken by two reviewers (RD and EvR) scoring 'relevant', 'doubt' or 'irrelevant' on forms. In case of inconsistencies, the reviewers discussed the scoring. Consensus on 'irrelevant' led to the exclusion of an article. On one occasion the reviewers asked for the involvement of a third party (LdW) in order to reach consensus. The same reviewers also performed independently the quality assessment of included studies as well as the data extraction. Inconsistencies in scoring between reviewers was discussed until consensus was reached. As the included trials all turned out to be randomized controlled trials the methodological quality was assessed using an adaptation of the Cochrane Back Review Group list of criteria (Table 2) [19]. Three items were disregarded, because of their use as an inclusion criterion (relevance of outcome measure) or their low applicability to the evaluated interventions (blinding of participant and blinding of care provider). The criteria list comprised five descriptive, two statistical and nine validity items. Each item was scored "+" if the criterion was fulfilled, "-" if the criterion was not fulfilled, and "?" if the information was not provided or was unclear. Scores on validity items ranged from 0 to 9 per trial. Trials which fulfilled over half of the validity items were considered to be of "sufficient methodological quality". In addition, general characteristics of the studies and outcomes of the trials were extracted. All data were analyzed qualitatively. Pooling of data was considered inappropriate due to the heterogeneity between trials regarding measurement instruments and intervention characteristics.

**Table 2:** Extracted data from the selected full papers

**1. Methodological Quality of Trials (Criteria List by Cochrane Back Review Group)**
*Descriptive Items*
1. Were eligibility criteria clearly specified?
2. Were index and control interventions explicitly described?
3. Was described whether adverse effects had or had not occurred?
4. Was a short-term follow-up measurement (directly after the intervention) of disability performed?
5. Was a long-term follow-up measurement (> 6 months after the intervention) of disability performed?

*Statistical Items*
6. Was the sample size for each group described?
7. Were point estimates and measures of variability presented for disability?

*Internal Validity Items*
8. Was a method of randomization used?
9. Was treatment allocation concealed?
10. Were groups similar at baseline regarding the most important prognostic indicators?
11. Were co-interventions avoided or comparable?
12. Was the compliance acceptable in all groups?
13. Was the outcome assessor blinded to the intervention?
14. Was the withdrawal/dropout rate acceptable (maximum of 20% for short-term follow-up and 30% for long-term follow-up)?
15. Was timing of the outcome assessment in both groups comparable?
16. Did the analysis include an intention-to-treat analysis?



**2. Results of the Trial**

Disability outcome measure
Frailty components measures
Baseline and follow-up details: number of participants, time of follow-up and results of the analyses

## Results

**Figure 1:**
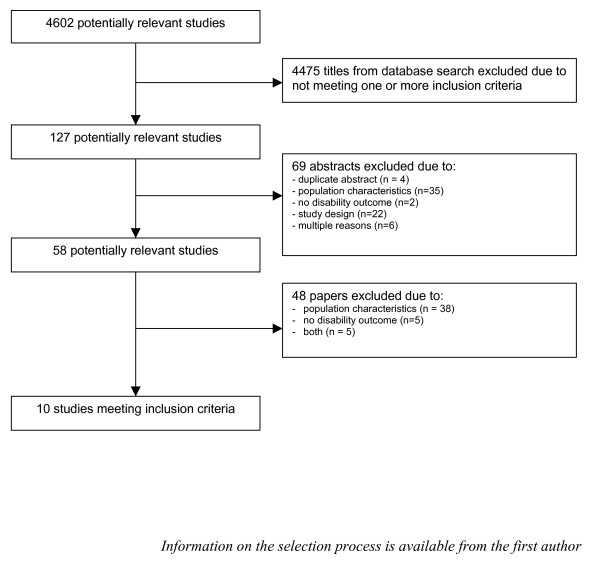
**Progress of search for relevant trials**.

Four thousand six hundred and two titles were identified in the literature search. After screening the titles, 127 studies were considered relevant for further screening on abstract-level. Of these, another 69 studies were excluded, because of not meeting the inclusion criteria (figure 1). In the next phase, the screening of 58 full-text articles resulted in the exclusion of 48 studies. Thirty-eight trials were excluded for not meeting the criteria for population characteristics, 5 for not meeting the criteria for the outcome measure (disability) and 5 for not meeting both criteria. There was a 0.70 and 0.75 (Kappa value) agreement between the reviewers during respectively screening of abstracts and screening of full-text articles. Results of the methodological quality assessment of the 10 included trials are shown in table 3. The observed total validity score ranged from 3 to 7. One trial [20]did not fulfill over half of the criteria (≥ 5) and was considered to be of insufficient methodological quality. Most papers did not provide sufficient information on whether the treatment allocation was concealed and on whether co-interventions were avoided or comparable. Concerning the descriptive items, 9 out of the 10 trials did not perform a long-term follow-up measurement (≥ 6 months after the intervention). Six trials did not report about the occurrence of adverse effects.

**Table 3:** Methodological quality of included trials*

	**Descriptive**^**†**^				**Statistical**				**Internal Validity**								**Total Validity**	
**Study**	**1**	**2**	**3**	**4**	**5**	**6**	**7**	**8**	**9**	**10**	**11**	**12**	**13**	**14**	**15**	**16**	**?**	**+**

Binder, 2002	+	+	+	+	-	+	+	+	+	+	?	?	+	-	+	+	2	6

Boshuizen, 2005	+	+	-	+	-	+	+	+	?	?	?	+	+	-	+	+	3	5

Chandler, 1998	+	+	-	+	-	+	-	+	?	+	?	?	+	+	+	+	3	6

Chin A Paw, 2001	+	+	+	+	-	+	+	+	?	+	?	+	-	-	+	+	2	5

Gill, 2002	+	+	+	+	-	+	+	+	?	+	?	+	+	+	+	+	2	7

King, 2002	+	+	+	+	-	+	+	+	?	+	?	-	+	-	+	+	2	5

Kretser, 2003	+	+	-	+	-	+	+	?	-	?	?	?	?	+	+	+	5	3

Payette, 2002	+	+	-	+	-	+	+	+	?	+	?	-	+	+	+	+	2	6

Timonen, 2004	+	+	-	+	+	+	-	+	?	+	?	+	?	+	+	+	3	6

Worm, 2001	+	-	-	+	-	+	-	+	-	?	?	+	?	+	+	+	3	5

* '+' criterion fulfilled; '-' criterion not fulfilled; and '?' data not provided or unclear.

^† ^see table 2 for a detailed description of the items.

Additional file 1 provides general characteristics of all 10 trials. From here the results of the 9 trials with sufficient methodological quality will be presented. The number of participants in the trials varied from 46 to 188. All studies were published between 1998 and 2005, showing the relative new trend in using frailty indicators explicitly as inclusion criteria. Disability was not the primary outcome measure for all studies. In the 9 trials the experimental interventions were nutritional interventions [21,22] or physical exercise interventions [21,23-29]. The study by Chin A Paw [21] followed a factorial design evaluating both a nutritional and a physical exercise program. The nutritional interventions in the selected studies were focused on macronutrient status [22] or micronutrient status [21]. The physical exercise interventions were single-component focusing purely on lower extremity strength [23,27] or multi-component addressing a variety of physical parameters as endurance, flexibility, balance and strength [21,24-26,28,29].

All interventions can be regarded as standard treatment (as in contrast to tailor-made treatment) and focused on the physical condition of the participants, except for one [26] that individualized treatment based on the outcomes of an extensive assessment and focused also on environmental conditions. The interventions lasted from 10 weeks to 18 months. The two longest programs [25,26] intended to encourage participants to undertake home exercising independently and provided only monthly phone calls in the last 6 months. In this review additional support by telephone was considered a part of the intervention.

The inclusion criteria used varied from rising from a chair, to descending stairs, knee extensor strength, oxygen-uptake, physical inactivity, involuntary weight loss, low BMI, dietary assessment, gait test, balance test and mobility problems (see also inclusion criteria in Additional file 1). Although all studies used measurements on ADL or IADL, not all mentioned disability as an outcome measure; some used other terms like functional decline [26] or reduced functional ability [24]. The mean age of the populations in the included trials ranged from 76 to 83.

The main results of the trials are presented in Additional file 1. All trials (except for one [28]) reported statistically significant positive changes on physical measures e.g. weight gain, strength, mobility, oxygen-uptake, physical fitness, physical activity and balance. Improvements on these physical measures did not necessarily lead to positive effects on disability outcomes, as only 3 out of nine trials reported significant differences in favor of the intervention group [24,26,29]. Those three trials are multi-component physical exercise programs. In the study of Gill et al. [26], the intervention group showed less functional decline at 12 months (from 2.3 to 2.7 on a scale with a range of 0 to 16) than the control group (from 2.8 to 4.2). Subgroup analysis revealed that these effects were mainly obtained for participants with moderate frailty but not for participants with severe frailty. Worm et al. [24] reported for the intervention group a larger increase in functional ability (from 36.4 to 53.6 on a scale ranging from 0 to 100) compared to the control group (from 39.1 to 43.0). In the study of Binder et al. [29] the intervention group reported less difficulty with ADL and IADL after the nine months program (from 26.6 to 30.4 on a scale ranging from 0 to 36) compared to the control group (from 26.6 to 27.0). Binder et al. [29] specifically focused on moderate to mild frail elderly.

## Discussion

This review aims to provide an overview of the content, methodological quality and effectiveness of intervention studies that are directed to physically frail elderly. In order to perform this review a strict use of criteria for physical frailty and disability was needed. This meant that even recent trials that contribute to frailty intervention research (e.g [30]), however not meeting the inclusion criteria, were excluded. The use of the term frailty in the literature is relatively new [4]. Although reference lists were checked, a limitation of this review is that 'older studies' were probably not identified as we searched with terms for frailty and synonyms. Another risk for publication bias is the selection of 4602 studies on title-level that may have resulted in excluding relevant articles. Due to a lack of consensus in the literature about measures for detecting frail elderly the homogeneity of the target groups in this review may have been reduced. In interpreting the results of this review it should be taken in consideration that due to small sample sizes trials may have been underpowered to detect differences on the self-reported measures for disability.

Out of the 10 randomized controlled trials that evaluated interventions for physically frail community-living elderly on disability, 9 were considered to be of sufficient methodological quality. No consistent findings were found in these 9 trials regarding their effect on disability.

There is no evidence that nutritional interventions for frail elderly, despite an observed effect on total energy intake and weight gain [22], result in positive effects on disability-level. Out of 8 physical exercise interventions three reported positive outcomes for disability. No evidence appeared that single lower extremity strength-training, despite the effect on strength [23,27] and walking function [27] has an effect on disability. However, lower extremity strength training as part of a multicomponent program may contribute to the effectiveness of these programs. Out of six trials that offer a multi-component physical exercise program focusing on endurance, flexibility, balance and strength, three studies [24,26,29] reported statistically significant effects for the disability outcome. The interventions in the effective studies of Gill et al. [26] and Binder et al. [29] are relatively long-lasting programs (respectively 12 and 9 months) with at least three exercising moments a week. The effective program of Worm et al. [24] last 12 weeks and comprises 2 supervised sessions a week and daily home exercises for 8 to 10 minutes. Gill et al.'s trial [26] has some specific features compared to the other trials: it is more individualized, it focuses on both the person and the environment and it provides supervised individual home-sessions followed by six months of non-supervised exercising.

The differences between the interventions hamper the interpretation of elements that consistently produced successful outcomes. Although malnutrition and physical frailty markers are considered strong indicators for functional decline in the elderly [31,32], the question is whether targeting underlying mechanisms of frailty can prevent or delay disability. The effects of single nutritional interventions on functional performance is an issue under discussion [31]. A review on protein and energy supplementation in malnourished elderly [33] reported evidence for weight gain, but found no evidence for positive effects on functional performance. Our finding that physical exercise interventions for community-dwelling frail elderly have an effect on intermediate physical measures is supported by reviews [34-36] focusing on physical exercising for elderly. These identified a lack of evidence for the effect of physical exercise on disability, suggesting that prevention of disability needs to address a complex of physical, behavioral, environmental and social factors. This review on interventions for physically frail elderly shows some indication that long-lasting high-intensive exercise programs for moderate physically frail elderly can have an effect on disability outcomes. However, additional research is needed. As there are also indications that especially moderate frail elderly may benefit [14,26] it is recommended to conduct subgroup analysis in effect studies for frail elderly.

## Conclusion

A systematic review was conducted to assess the content, the methodological quality and the effectiveness of intervention studies for the prevention of (ADL/IADL) disability in community-dwelling physically frail older persons. There is no evidence that nutritional interventions for frail older persons, despite an observed effect on total energy intake and weight gain, result in positive effects on disability-level. No evidence appeared that single lower extremity strength-training, despite the effect on strength and walking function, has an effect on disability for physically frail older persons. There is some indication that long-lasting high-intensive exercise programs for moderate physically frail older persons can have an effect on disability outcomes.

## Competing interests

The authors declare that they have no competing interests.

## Authors' contributions

RD, EvR, LdW and WvdH developed the original idea for the review. RD performed the search strategy and the selection, extracted the data and wrote the manuscript. EvR was involved in the selection process and data extraction. EvR, LdW, WvdH and GIJMK provided valuable comments during the process of writing this manuscript. All authors have read and approved the final manuscript.

## Pre-publication history

The pre-publication history for this paper can be accessed here:



## Supplementary Material

Additional file 1 **Table 4: General characteristics and outcomes of included trials.** The data provided informs about features of interventions, measurements and outcomes of trials included in the systematic review Click here for file
