# Supplementary material for: Interventions to prevent disability in frail community-dwelling elderly: a systematic review
Source: BMC Health Serv Res. 2008 Dec 30;8:278. doi: 10.1186/1472-6963-8-278 (PMC2630317; doi:10.1186/1472-6963-8-278)
Supplement: Additional file 1 — Table 4: General characteristics and outcomes of included trials. The data provided informs about features of interventions, measurements and outcomes of trials included in the systematic review [file 1472-6963-8-278-S1.doc]

| Table 4. General Characteristics and Outcomes of Included Trials | | | | | | | | | | | | | | |
| --- | --- | --- | --- | --- | --- | --- | --- | --- | --- | --- | --- | --- | --- | --- |
| Study | NRandomized/Followed-up^*^ | | | Participants **Inclusion criteria and mean age** | | Interventions | | Primarily aimed at^†^ | | Follow-up^‡^ | | **Outcome**  **measures§** | | **Results** ^║^ |
| Binder, 2002  USA |  | IG:  69/66  CG:  50/49 | Two out of three criteria: score between 18-32 on Modified PPT, peak oxygen uptake between 10-18 mL/kg/min, self-reported difficulty in max. 2 ADLs or 1 IADL  (Mean age: 83) | | *IG*: 9-month program provided by physiology exercise technicians consisting of three phases of each 36 sessions. Phase 1: Group format: 22 exercises on flexibility, balance, coordination, speed of reaction and strength. Phase 2: Progressive resistance training combined with shortened version of phase 1 exercises. Phase 3: Endurance training combined with shortened version of phase 1 and phase 2 exercises.  *CG*: 9-month home exercise program including nine of the 22 exercises from phase 1. Participants were asked to perform exercises 2-3 times a week and attended a monthly class. | | Reduce or delay frailty | | 3, 6 and 9 months | | - Self-reported use of assistance or assistive technology (OARS ADL and IADL scale) - Self-reported difficulty with ADL and IADL (Physical Function subscale of FSQ) - Modified PPT items - Knee extension /flexion strength - V02-peak - Balance - Body weight | | No differences between groups (data not presented).  Difference (SS) in favor of IG.  Differences (SS) in favor of IG for modified PPT score, knee strength, V02-peak and balance. No SS difference for body weight (data not shown). | |
| Boshuizen, 2005, Netherlands |  | IG: high guidance (HG)  24/16  IG: medium guidance (MG)  26/16  CG:  22/17 | Experiencing difficulty in rising from chair and maximum knee extensor torque <87.5 N-m  (Mean age:~79) | | *IG: High-guidance*: 10-wk exercise program, each week two group sessions (60 min) by PT and one unsupervised home session. Focus on exercises with a variation in concentric, isometric and eccentric knee-extensor activity.  *IG: Medium-guidance*: same program, though each week one supervised session and two unsupervised home sessions.  *CG:* No training or other encouragement | | Increasing strength knee  extensors | | 10-12 weeks | | - Self-reported performance in activities of daily living (GARS) - Knee extensor strength - Walking function - Balance - Box stepping - Timed Up and GO | | No differences between groups.  Difference (SS) in knee strength and walking function for HG group compared to CG. Differences (NS)  between HG and MG for walking function in favor of HG. | |
| Chandler, 1998, USA |  | IG:  50/44  CG:  50/43 | Inability to descend stairs step over step without holding the railing  (Mean age: ~78) | | *IG:* 10-wk exercise program, each week three individual in-home sessions by PT. Focus: progressive resistive lower extremity exercises with theraband.  *CG:* No training or any encouragement. | | Increasing lower  extremity strength | | 10 weeks | | - Self-reported limitations in physical activities (MOS-36 physical functioning subscale) - Lower extremity strength - Physical   performance | | No significant differences  between groups (data not presented).  Greater lower extremity (SS) strength gain in favor of IG (data on physical performance not presented). | |
| Chin A Paw, 2001, Netherlands |  | IG a:  39/39  IG b:  39/39  IG c:  42/42  CG:  37/37 | Physical inactivity and involuntary weight loss or body mass index below 25kg/m²  (Mean age:~79) | | *IG a:* 17- wk exercise program, each week 2 group sessions (45 min) by experienced instructors. Focus: skills training focused on learning physiologic parameters to perform and sustain motor action.  *IG b:* 17- wk nutrition program; several fruit and dairy products enriched with vitamins and minerals. One fruit and a diary product in addition to daily diet or as a replacement.  *IG c*: 17-wk both exercise and nutrition program.  *CG:* No exercise or nutrition or any other kind of encouragement.  (note: this trial followed a factorial design for studying the effect of nutrition and the effect of exercise) | | Maintenance or improvement  mobility and performance  Improvement of  micro-nutrient  intake and status | | 17 weeks | | - Self-rated disabilities (name instrument not specified) - Functional capacity - Physical fitness - Physical activity | | No difference between  exercise groups (a+c) and non-exercise groups (b+d) was found.  No difference between  enriched food groups (b+c) and regular food groups (a+d) was found.  Difference (SS) in favor of exercise group (a+c) vs non-exercise groups (b+d) for functional capacity and fitness score.    No differences between enriched food groups (b+c) and regular food groups (a+d) were found for functional capacity, physical fitness and physical activity. | |
| Gill, 2002, USA |  | IG:  94/88  CG:  94/90 | Aged ≥75; performance on rapid gait test >10 seconds or inability to stand up from seated position  (Mean age: ~83) | | *IG:* 12- month program with average of 16 visits (46-60 min) by PT and home exercising in the first 6 months, followed by 6 phone calls (by PT) in the next 6 months to encourage further (daily) home exercising. Focus: instruction in safe techniques, proper use of devices, removal of environmental hazards and exercises for range of motion, balance, muscle conditioning and strengthening.  *CG:* 12-month educational program with six monthly home visits and six additional monthly phone calls by a health educator promoting good health (e.g. nutrition, management of medications, physical activity, sleep hygiene). | | Prevention of functional decline | | 3, 7 and 12 months | | - Self-reported performance in activities of daily living (name instrument not specified) - Mobility - Physical performance (as reported in[37]) | | Differences (SS) between groups in favor of IG.  Differences (SS) in favor of IG in both measures. | |
| King, 2002, USA |  | IG:  80/45  CG:  75/54 | Aged ≥70; SPPB score ≤ 9 AND independence in at least 5 ADL’s  (Mean age: ~77) | | *IG:* 18 month-exercise program with 6 months of 3 group sessions (75 min) each week (by PT and/or exercise leader), followed by 6 months of 1 group session a week and two home-exercise sessions, and 6 months of 3 home-exercises a week with monthly call from exercise leader. Focus; training on endurance, strength, balance and flexibility.  *CG:* 18 month-program with one instructional session on principles of physical activity, exercise and nutrition.  A booklet was given recommending 180 minutes of exercising a week. Phone calls each month over 18 months-period to encourage completion of exercise logs. | | Increasing strength, endurance, mobility and flexibility | | 3, 6, 12 and 18 months | | - Self-reported limitations in physical activities (SF-36 physical functioning subscale) - Physical performance - Walking function - Mac Arthur (MA) battery - Balance | | Scores for both IG and CG improved during 18 months. At 6 and 12 months there is slight difference (NS) in favor of the CG. At 18 months, slight difference (NS) in favor of the IG.  At 3 months: improvement (SS) in MA battery in favor of IG. At 6 months: improvement (SS) in chair rise, standing balance, MA battery and gait speed in favor of IG. At 12 months: difference (SS) for MA battery, gait speed and standing balance in favor of IG.  At 18 months: no SS between groups for any measure. | |
| Kretser, 2003, USA |  | IG:  102/83  CG:  101/80 | Score <22.5 on Mini Nutritional Assessment (MNA)  (Mean age: ~76) | | *IG:* 6-month nutrition program consisting of weekly 21 meals and 14 snacks meeting 100% of the Daily Reference Intake (DRI) needed for people over 50 and with a daily caloric level of 1800 kcal.  *CG:* 6-month nutrition program consisting of delivery of 5 hot meals per week meeting 33% of DRI. | | Improving weight and functionality | | 6 months | | - (Self?) Reported performance of activities of daily living (name instrument not specified) - MNA scores - Weight gain | | No differences between groups. Subgroup analysis show a slight difference (NS) in malnourished participants for IADL-decline in favor of IG.  Weight gain (SS) for IG. | |
| Payette, 2002, Canada |  | IG:  42/42  CG:  41/39 | Aged >65; involuntary weight loss and BMI <27 or BMI<24  (Mean age: ~80) | | *IG:* 16-wk nutrition program; provision of 235-ml cans of nutrient-dense protein-energy liquid. Two cans a day in addition to usual diet. Each month 1 home visit and each two weeks a phone call was conducted by a dietician.  *CG:* No treatment, though participants were visited each month and received a small gift. | | Improving nutritional status and reverse weight loss | | 16 weeks | | - Self-reported limitations in role functioning due to physical health and due to emotional problems (SF-36 physical role functioning scale and emotional role functioning scale) - Energy intake - Weight gain - Anthropometric indexes - Strength - Timed Up and Go | | No SS differences between groups. Both groups show improvement on Physical Role Functioning. Differences (NS) in favor of IG is seen for Emotional Role Functioning.  Differences (SS) for total energy intake and weight gain in favor of IG. No SS differences on other measures. | |
| Timonen, 2004, Finland |  | IG:  34/32  CG:  34/32 | Aged≥75; difficulty with balance and mobility and symptoms of dizziness or reported falls or difficulty in walking independently  (Mean age: ~83) | | *IG:* 10-wk exercise program, each week two group sessions (90 min) supervised by two PT’s. Core of program; progressive resistance training (lower extremity) and functional exercises. One home visit (PT) to instruct stretching program to be performed 2-3 times a week at home .  *CG:* One home visit by PT, who taught a home exercise program. Subjects were advised to perform two sets of 15 repetitions 2-3 times a week. No further encouragement to exercise was given. | | Increasing strength and functional status | | 1 week, 3 months and 9 months | | - Performance of activities of daily living based on interviews and observations (Joensuu Classification Model) - No other outcome measures were used | | No SS differences between groups. | |
| Worm, 2001, Denmark |  | IG:  22/21  CG:  24/23 | Aged ≥74;inability to leave home unaided or unattended or without mobility aids  (Mean age: ~81) | | *IG:* 12-wk exercise program with 2 group sessions per week(60 min); flexibility training, aerobics and rhythm, balance and reaction exercises, muscle training. Participants were also asked to perform a home-based exercise program (8-10 min) every morning.  *CG:* No treatment or any other kind of encouragement. | | Improving balance, endurance, muscle strength, coordination and reaction capacity | | 12 weeks | | - Self-reported limitations in physical activities (SF-36 physical functioning subscale) - Balance - Walking function - Muscle strength shoulder - Aerobic capacity | | Differences (SS) between groups in favor of IG.  Differences (SS) in balance, walking function and muscle strength in favor of IG. | |

*Randomized relates to the number of participants randomized to intervention (IG) and control group (CG). Followed-up relates to the numbers of participants taken into data-analysis

^†^ Outcome which the authors primarily aimed to improve by conducting the intervention.

^‡^ Follow-up measurement in weeks or months after randomization.

^§^ Measured disability concept and instrument, followed by outcome measures for frailty components;

^║^ SS = statistically significant difference if p < 0.05: NS = not statistically significant
